# Correlation between previous sedentary lifestyle and CrossFit-related injuries

**DOI:** 10.31744/einstein_journal/2021AO5941

**Published:** 2021-04-28

**Authors:** Tiemi Maruyama de Moura Paiva, Michel Kanas, Nelson Astur, Marcelo Wajchenberg, Delio Eulalio Martins

**Affiliations:** 1 Hospital Israelita Albert Einstein Faculdade Israelita de Ciências da Saúde Albert Einstein São PauloSP Brazil Faculdade Israelita de Ciências da Saúde Albert Einstein, Hospital Israelita Albert Einstein, São Paulo, SP, Brazil.; 2 Hospital Israelita Albert Einstein São PauloSP Brazil Hospital Israelita Albert Einstein, São Paulo, SP, Brazil.

**Keywords:** Crossfit, Wounds and injuries, Athletes, Sedentary behavior, Exercise

## Abstract

**Objective:**

To correlate CrossFit-related injuries with previous sedentary lifestyle, and to investigate other factors potentially associated with higher rates of injury among practitioners.

**Methods:**

A nationwide cross-sectional study involving CrossFit practitioners who received a digital questionnaire inquiring into CrossFit-related injuries, previous sedentary life, training intensity and experience, site of injury and general demographics.

**Results:**

This sample included 121 CrossFit practitioners, 34.7% of participants were sedentary prior to starting CrossFit practice, from these, 45.2% reported CrossFit-related injuries, compared to 30.4% from previously active practitioners (p=0.104). The shoulder/elbow (60.5%), lumbar spine (30.3%) and wrist/hand (16.3%) were the most common sites of injury among participants reporting CrossFit-related injuries (35.5%). Participants performing intense weight training were more prone to injuries than those practicing light or moderate weight training (p=0.043). On average, participants with a history of injury spent significantly more time training than those with no history of injury (68.4 and 61.7 minutes, respectively; p=0.044).

**Conclusion:**

The incidence of CrossFit-related injuries did not differ significantly between previously sedentary and physically active participants. Intense weight training was associated with a higher incidence of injuries. The overall injury rate was 35.5%, similar to that found in previous studies, and the most common site of injury was shoulder/elbow.

## INTRODUCTION

CrossFit is a strengthening and conditioning program based on high intensity functional movement created by Glassman in 2000.^(^^1^^)^ Increasing popularity of this fitness program worldwide has sparkled the interest of physically active adults.^(^^2^^)^ The huge growth in (the number of CrossFit practitioners raised concerns about potential practice-related injuries.^(^^3^^,^^4^^)^

Higher incidence of shoulder, spinal and knee injuries has been reported.^(^^5^^–^^7^^)^ However, no evidence that CrossFit is associated with a higher incidence of injuries relative to other sports has been given so far and the rate of CrossFit-related injuries (0.74 to 3.3 per 1,000 hours of training) is similar to sports such as weightlifting, gymnastics and tennis.^(^^5^^,^^8^^,^^9^^)^ Risk factors for higher incidence of CrossFit-related injuries are athletic skills, hours of training, participation in competitions and trainer supervision.^(^^5^^–^^7^^)^

CrossFit has appealed to physically active as well as sedentary individuals. Sedentary lifestyle prior to committing to a CrossFit program has been pointed out as a potential risk factor for higher incidence of injuries.^(^^2^^,^^10^^)^

## OBJECTIVE

To correlate CrossFit-related injuries with previous sedentary lifestyle, and to investigate other factors potentially associated with higher rates of injury among practitioners.

## METHODS

This study was approved by the institutional review board (CAAE: 90062918.7.0000.0071, No. 2.805.401). CrossFit practitioners selected out of a list of certified CrossFit centers in Brazil were contacted by e-mail between August and December 2018. An Informed Consent Form (ICF) was sent and access to a digital questionnaire granted to eligible practitioners. To be eligible, practitioners had to be enrolled in a CrossFit program and practicing for at least 6 months.

Participants were asked whether they were physically active or sedentary prior to enrollment in a CrossFit program, whether they had sustained any CrossFit-related injuries and, if so, at which site. Injury was defined as any condition requiring training modification or discontinuation, or visit to a health professional for treatment or diagnosis. Data such as time and volume of practice, competitive background and training intensity (weight, speed and frequency) were gathered. General data, such as demographics and body mass index (BMI), were also collected.

Statistical analysis of sample characteristics and questionnaire questions was based on descriptive statistics, including mean, range and standard deviation (SD). Correlations between previous sedentary lifestyle and CrossFit-related injuries were investigated using the χ^2^ test. Categorical variables were compared using the χ^2^ or the Fisher’s exact test. Numerical variables were compared using the Mann-Whitney test. Injury rates were described with 95% confidence intervals. P-values lower than 0.05 were considered significant. Statistical tests were performed using software R, version 3.0.3 (R Foundation for Statistical Computing, Vienna, Austria).

## RESULTS

This sample comprised 121 CrossFit practitioners who agreed to participate in the study, signed the ICF and answered the digital questionnaire. Demographic characteristics of participants are described in table 1. Most participants were female (56.2%) and mean BMI was 24.34.

**Table 1:** Demographic characteristics of participants and training intensity data

Variable		
Sex		
	Female	68 (56.2)
	Male	53 (43.8)
Height, m		1.70±0.10
Weight, kg		70.91±15.07
BMI		24.34±3.40
Were you physically active before you started practicing CrossFit?		
	No	42 (34.7)
	Yes	79 (65.3)
How long have you been practicing CrossFit or for how long have you practiced?		
	Less than 1 month	1 (0.8)
	1 to 3 months	6 (5.0)
	3 to 6 months	14 (11.6)
	6 months to 1 year	27 (22.3)
	More than 1 year	73 (60.3)
Have you participated in official CrossFit competitions?		
	No	81 (66.9)
	Yes	40 (33.1)
How intense is your practice? (weight)		
	Light	8 (6.6)
	Moderate	71 (58.7)
	Intense	42 (34.7)
How intense is your practice? (speed)		
	Light	18 (14.9)
	Moderate	70 (57.9)
	Intense	33 (27.3)
How intense is your practice? (frequency)		
	Light	5 (4.1)
	Moderate	44 (36.4)
	Intense	72 (59.5)

Results expressed as n (%) or mean±standard deviation.

BMI: body mass index.

Overall, 65.3% of the participants described themselves as physically active (*i.e.*, they exercised regularly) before starting a CrossFit program, whereas 34.7% declared being sedentary prior to practicing CrossFit. Most participants (60.3%) had been practicing for more than one year.

Most participants (64.5%) declared not having sustained any injuries. The shoulder/elbow (60.5%), lumbar spine (30.3%) and wrist/hand (16.3%) were the most common sites of injury among participants with a history of CrossFit-related injuries (35.5%) (Table 2).

**Table 2:** Data on CrossFit-related injuries

Questions		n (%)
Have you had any injuries associated with CrossFit practice?, n=121		
	No	78 (64.5)
	Yes	43 (35.5)
Knee injury		3 (7.0)
Ankle/foot injury		6 (14.0)
Hip injury		1 (2.3)
Lumbar spine injury		13 (30.3)
Cervical spine injury		4 (9.3)
Shoulder/elbow injury		26 (60.5)
Wrist/hand injury		7 (16.3)
Other types of injuries		6 (14.0)
How long did you have to interrupt training due to injury?, n=43		
	1-2 days	3 (7.0)
	3-5 days	7 (16.3)
	1 week	4 (9.3)
	2 weeks	14 (32.6)
	1 month	5 (11.6)
	More than 1 month	8 (18.6)
	Permanently	2 (4.7)
Was there a need to modify the duration, intensity or usual training regimen for more than 2 weeks?, n=43		
	No	11 (25.6)
	Yes	32 (74.4)
Have you sought medical attention after the injury?, n=43		
	No	12 (27.9)
	Yes	31 (72.1)

Correlations between history of sedentarism and CrossFit-related injuries were investigated using the χ^2^ test. Injury rates did not differ significantly between previously sedentary and physically active participants (p=0.104) (Table 3). However, the percentage of injuries was higher among participants reporting previous sedentary lifestyle (45.2% *versus* 30.4%).

**Table 3:** Correlations between previous practice of physical activity and injury

Lifestyle prior to CrossFit practice	No injury (%)	Injured (%)	p value*
Sedentary lifestyle	23 (29.5)	19 (44.2)	0.104
Physically active	55 (70.5)	24 (55.8)	

* p value obtained in the χ^2^ test for comparison of the groups.

Training intensity data were classified into three domains (weight, speed and frequency). The practice was described as intense for weight, speed or frequency, by 34.7%, 27.3% and 59.5% of participants respectively. Analysis of correlations between training intensity and rate of injury revealed significant differences between participants with and without a history of injury in the weight domain. Participants who did intense weight training had a higher incidence of injuries compared with those practicing light or moderate intensity (p=0.043) (Table 4). There were no significant differences in the speed and frequency domains.

**Table 4:** Correlations between CrossFit training intensity and injury

CrossFit training intensity		No injury (%)	Injured (%)	p value
How intense is your training? (weight)				0.043
	Light or moderate	56 (71.8)	23 (53.5)	
	Intense	22 (28.2)	20 (46.5)	
How intense is your training? (speed)				0.068
	Light or moderate	61 (78.2)	27 (62.8)	
	Intense	17 (21.8)	16 (37.2)	
How intense is your training? (frequency)				0.088
	Light or moderate	36 (46.2)	13 (30.2)	
	Intense	42 (53.8)	30 (69.8)	

The majority of participants (58.1%) who reported having sustained an injury were male (p=0.018). On average, participants with a history of injury spent significantly more time training than those with no history of injury (68.4 and 61.7 minutes respectively, p=0.044).

## DISCUSSION

CrossFit popularity has increased substantially and so has the concern with practice-related injuries. with no previous experience with physical activities, or clearly stating a sedentary lifestyle, are also signing into its program. In this study, we hypothesized these specific populations would be more prone to CrossFit-related injuries. However, injury rates did not differ significantly between these and physically active participants. Given CrossFit involves complex movements, lack of prior conditioning is an obvious concern. Previously sedentary participants did in fact sustain more injuries than physically active ones, but differences were non-significant. This was probably due to the small size of this sample. In a study comparing related injuries between previously sedentary and active practitioners, Sprey et al.,^(^^2^^)^ also found injury rates to be very similar (30.4% and 31.2%, respectively). A recent prospective cohort study^(^^9^^)^ investigating the incidence of injuries among beginner practitioners enrolled in an eight-week CrossFit program revealed a higher risk (14.9%) of injuries in this population relative to experienced practitioners.

CrossFit-related injuries are mostly caused by muscle overload during practice. Overload injuries result from a high number of repetitions of the sporting gesture, lifting weights beyond one’s capability or any other activity that exceeds the physiologic limit of the participant.^(^^11^^–^^13^^)^ Hopkins et al.,^(^^14^^)^ reported 11 cases of rhabdomyolysis after CrossFit practice. CrossFit is thought to be associated with a higher incidence of injuries. Still, previous studies revealed a similar incidence to running, tennis, gymnastics and Olympic weightlifting and a much lower incidence relative to rugby and soccer.^(^^2^^–^^5^^,^^7^^,^^8^^)^ As in other studies,^(^^2^^,^^5^^,^^15^^,^^16^^)^ the incidence of injuries in this study was 35.5%. However, in a recent prospective study, the rate was 14.9%.^(^^9^^)^ Variations in the incidence of injuries between studies are likely due to differences in the definition of injury adopted or the profile of participants.

The spine, shoulder and knee are the most common sites of CrossFit-related injuries.^(^^5^^,^^7^^,^^8^^,^^14^^,^^15^^,^^17^^)^ In a study conducted by Hopkins et al., the spine was the most common site of injuries. In that study, spinal injuries accounted for 20.9% of injuries overall and 6.7% of cases required surgical treatment. CrossFit involves similar sporting gestures to activities such as gymnastics and weightlifting; therefore, injury patterns and sites are very similar.^(^^14^^)^ In this study, shoulder/elbow were the primary site of injuries (60.5%), followed by the spine (39.6% adding lumbar and cervical) whereas the incidence of knee injury was low (7%). According to Sugimoto et al.,^(^^18^^)^ the chances of sustaining lower limb injuries is 2.65 times higher in women than in men. In contrast, shoulder injuries are 2.79 more likely in men, while spinal injuries are more common in practitioners aged under 19 years.

Male gender, long training sessions, training intensity, participation in competitions, high BMI, lack of supervision during training and previous injuries are some of the risk factors for CrossFit-related injuries.^(^^2^^,^^5^^–^^8^^)^ These data are consistent with findings of this study. In this sample, intense training in terms of frequency and weight were associated with higher rates of injury, but only associations in the weight domain was significant. Participants who had sustained injuries spent more time training (68.4 minutes) than injury-free participants. Injuries were also more common in males than in females.

The major limitation of this study was the relatively small sample size for statistical analysis. This probably reflected the reluctance of some CrossFit gyms to participate in this research and share the digital questionnaire with their athletes. Cross-sectional design was another limitation, especially due to the subjective perception of respondents of what the term injury actually means, even though the definition of injury was emphasized in the questionnaire. Digital questionnaires have the advantage of being easily shared and providing wider coverage for research purposes. However, they are subject to respondents’ interpretation.

## CONCLUSION

The incidence of CrossFit-related injuries did not differ significantly between previously sedentary and physically active participants. Intense weightlifting was associated with a higher incidence of injuries.
