# Supplementary material for: Correlation between previous sedentary lifestyle and CrossFit-related injuries
Source: Einstein (Sao Paulo). 2021 Apr 28;19:eAO5941. doi: 10.31744/einstein_journal/2021AO5941 (PMC8081438; doi:10.31744/einstein_journal/2021AO5941)
Supplement: Translation

# Translation: Correlação entre sedentarismo prévio e lesões relacionadas ao CrossFit

**Authors:** Tiemi Maruyama de Moura Paiva, Michel Kanas, Nelson Astur, Marcelo Wajchenberg, Delio Eulalio Martins

**Affiliations:** 1 São PauloSP Brasil Faculdade Israelita de Ciências da Saúde Albert Einstein, Hospital Israelita Albert Einstein, São Paulo, SP, Brasil.; 2 São PauloSP Brasil Hospital Israelita Albert Einstein, São Paulo, SP, Brasil.

**Keywords:** Crossfit, Ferimentos e lesões, Atletas, Comportamento sedentário, Exercício físico

## Abstract

**Objetivo:**

Correlacionar as lesões durante a prática do CrossFit com sedentarismo prévio e pesquisar outros fatores possivelmente relacionados ao aumento da taxa de lesão entre os praticantes.

**Métodos:**

Estudo transversal nacional, envolvendo praticantes de CrossFit, que receberam questionário digital para avaliar lesões relacionadas à prática da modalidade, à vida sedentária anterior, a intensidade e tempo de treinamento, à localização da lesão e à demografia geral.

**Resultados:**

Esta amostra incluiu 121 praticantes de CrossFit, 34,7% dos participantes eram sedentários antes de iniciar o CrossFit, desses, 45,2% sofreram alguma lesão relacionada a essa atividade, *versus* 30,4% dos que eram anteriormente ativos (p=0,104). Dos praticantes avaliados, 35,5% declararam história de alguma lesão relacionada ao CrossFit. Os locais mais frequentes foram ombro e cotovelo (60,5%), coluna lombar (30,3%), e punho e mão (16,3%). Os participantes que realizam levantamento de peso de forma intensa estiveram mais propensos a lesões do que aqueles que realizavam treinamento leve ou moderado (p=0,043). Os participantes com história de lesão apresentaram tempo médio de treinamento significativamente maior quando comparados àqueles sem antecedente de lesão (68,4 minutos *versus* 61,7 minutos; p=0,044).

**Conclusão:**

Não houve diferença significativa na incidência de lesões relacionadas ao CrossFit entre participantes previamente sedentários e fisicamente ativos. O levantamento intenso de pesos esteve relacionado à maior incidência de lesões. A taxa média de lesões encontradas neste estudo foi de 35,5%, semelhante a encontrada em estudos prévios. O local mais frequente foi ombro/cotovelo.

## INTRODUÇÃO

O CrossFit é um programa de fortalecimento e condicionamento baseado em movimentos funcionais de alta intensidade, criado por Glassman, em 2000.^(^^1^^)^ A popularidade mundial crescente desse programa de exercícios despertou o interesse de adultos fisicamente ativos.^(^^2^^)^ O grande aumento do número de praticantes do CrossFit trouxe à tona a preocupação com a possibilidade de lesões relacionadas à prática.^(^^3^^,^^4^^)^

Incidências mais altas de lesões de ombro, coluna e joelho foram relatadas.^(^^5^^–^^7^^)^ Entretanto, até hoje, não há evidências de que a incidência de lesões seja maior no CrossFit do que em outros esportes, e o índice de lesões decorrentes do CrossFit (0,74 a 3,3 por 1.000 horas de treino) se assemelha ao relatado em esportes como levantamento de peso, ginástica e tênis.^(^^5^^,^^8^^,^^9^^)^ Os fatores de risco para maior incidência de lesões são a habilidade atlética, o número de horas de treino, a participação em competições e a supervisão do treinamento.^(^^5^^–^^7^^)^

O CrossFit atraiu o interesse tanto de indivíduos fisicamente ativos como de indivíduos sedentários. O estilo de vida sedentário antes da prática do CrossFit foi apontado como possível fator de risco para a incidência mais elevada de lesões relacionadas.^(^^2^^,^^10^^)^

## OBJETIVO

Correlacionar as lesões durante a prática do CrossFit com sedentarismo prévio e pesquisar outros fatores possivelmente relacionados ao aumento da taxa de lesão entre os praticantes.

## MÉTODOS

Este estudo foi aprovado pelo Comitê Institucional de Revisão (CAAE: 90062918.7.0000.0071, número: 2.805.401). Praticantes de CrossFit selecionados de uma lista de centros certificados de CrossFit no Brasil foram contatados por *e-mail* entre agosto e dezembro de 2018. Os praticantes elegíveis receberam um Termo de Consentimento Livre e Informado (TCLI) e foram autorizados a acessar um questionário digital. As condições para elegibilidade foram estar matriculado em um programa de CrossFit e ter, no mínimo, 6 meses de prática.

Os participantes foram questionados a respeito do nível de atividade física antes da matrícula em um programa de CrossFit (fisicamente ativo ou sedentário), da história de lesões relacionadas ao CrossFit e, em caso afirmativo, qual o local da lesão. O termo “lesão” foi definido como qualquer condição que determinasse a necessidade de modificação ou interrupção do treinamento, ou de consulta com um professional da saúde para tratamento ou diagnóstico. Dados como tempo e volume de prática, experiência em competições e intensidade de treinamento (peso, velocidade e frequência) foram coletados, além de dados gerais, como características demográficas e índice de massa corporal (IMC).

A análise estatística das características da amostra e perguntas do questionário foi baseada na estatística descritiva, incluindo média, intervalo e desvio-padrão (DP). As correlações entre estilo de vida sedentário prévio e lesões relacionadas ao CrossFit foram investigadas por meio do teste do χ^2^. As variáveis categóricas foram comparadas empregando-se o teste do χ^2^ ou o teste exato de Fisher. As variáveis numéricas foram comparadas empregando-se o teste de Mann-Whitney. Os índices de lesões foram descritos com intervalos de confiança de 95%. Valores de p<0,05 foram considerados significantes. Os testes estatísticos foram realizados por meio de software R, versão 3.0.3 (R *Foundation for Statistical Computing*, Viena, Áustria).

## RESULTADOS

Esta amostra incluiu 121 praticantes de CrossFit que concordaram em participar do estudo, assinaram o TCLI e responderam ao questionário digital. As características dos participantes encontram-se descritas na tabela 1. A maioria dos participantes era do sexo feminino (56,2%) e o IMC médio foi de 24,34.

**Tabela 1:** Características demográficas dos participantes e dados referentes à intensidade do treinamento

Variável		
Sexo		
	Feminino	68 (56,2)
	Masculino	53 (43,8)
Altura, m		1,70±0,10
Peso, kg		70,91±15,07
IMC		24,34±3,40
Você era fisicamente ativo antes de começar a praticar CrossFit?		
	Não	42 (34,7)
	Sim	79 (65,3)
Há quanto tempo você pratica CrossFit ou por quanto tempo praticou?		
	Menos de 1 mês	1 (0,8)
	1-3 meses	6 (5,0)
	3-6 meses	14 (11,6)
	6 meses a 1 ano	27 (22,3)
	Mais de 1 ano	73 (60,3)
Você já participou de competições oficiais de CrossFit?		
	Não	81 (66,9)
	Sim	40 (33,1)
Qual a intensidade de sua prática? (peso)		
	Leve	8 (6,6)
	Moderada	71 (58,7)
	Intensa	42 (34,7)
Qual a intensidade da sua prática? (velocidade)		
	Leve	18 (14,9)
	Moderada	70 (57,9)
	Intensa	33 (27,3)
Qual a intensidade da sua prática? (frequência)		
	Leve	5 (4,1)
	Moderada	44 (36,4)
	Intensa	72 (59,5)

Resultados expressos como n (%) ou média±desvio-padrão.

IMC: índice de massa corporal.

Do total de participantes, 65,3% se descreveram como fisicamente ativos (prática regular de exercício) antes de iniciar o programa de CrossFit, enquanto 34,7% se declararam sedentários antes de praticar CrossFit. A maioria dos participantes (60,3%) já vinha praticando há mais de 1 ano.

A maioria dos participantes (64,5%) declarou não ter sofrido lesões. Os locais mais comuns de lesão entre os participantes com história de lesões relacionadas ao CrossFit (35,5%) foram o ombro/cotovelo (60,5%), a coluna lombar (30,3%) e o punho/mão (16,3%) (Tabela 2).

**Tabela 2:** Dados referentes às lesões relacionadas ao CrossFit

Perguntas		n (%)
Você já sofreu lesões relacionadas à prática do CrossFit?, n=121		
	Não	78 (64,5)
	Sim	43 (35,5)
Lesão de joelho		3 (7,0)
Lesão de tornozelo/pé		6 (14,0)
Lesão de quadril		1 (2,3)
Lesão de coluna lombar		13 (30,3)
Lesão de coluna cervical		4 (9,3)
Lesão de ombro/cotovelo		26 (60,5)
Lesão de punho/mão		7 (16,3)
Outros tipos de lesões		6 (14,0)
Por quanto tempo você teve que interromper o treinamento por estar lesionado?, n=43		
	1-2 dias	3 (7,0)
	3-5 dias	7 (16,3)
	1 semana	4 (9,3)
	2 semanas	14 (32,6)
	1 mês	5 (11,6)
	Mais de 1 mês	8 (18,6)
	Permanentemente	2 (4,7)
Foi necessário modificar a duração, a intensidade ou o regime usual de treinamento por mais de 2 semanas?, n=43		
	Não	11 (25,6)
	Sim	32 (74,4)
Você buscou atenção médica após a lesão?, n=43		
	Não	12 (27,9)
	Sim	31 (72,1)

As correlações entre a história de sedentarismo e lesões relacionadas ao CrossFit foram investigadas por meio do teste do χ^2^. O índice de lesões não diferiu de forma significante entre os participantes com história de sedentarismo e os que eram fisicamente ativos (p=0,104) (Tabela 3). Entretanto, a percentagem de lesões foi mais alta entre os que relataram estilo de vida sedentário prévio (45,2% *versus* 30,4%).

**Tabela 3:** Correlações entre prática prévia de atividade física e lesões

Estilo de vida antes da prática do CrossFit	Sem lesões (%)	Com lesões (%)	Valor de p*
Estilo de vida sedentário	23 (29,5)	19 (44,2)	0,104
Fisicamente ativo	55 (70,5)	24 (55,8)	

* Valor de obtido no teste do χ^2^ para comparação entre grupos.

Os dados referentes à intensidade do treinamento foram classificados de acordo com três domínios (peso, velocidade e frequência). A prática foi descrita como intensa em termos de peso, velocidade ou frequência, por 34,7%, 27,3% e 59,5% dos participantes, respectivamente. A análise das correlações entre a intensidade do treinamento e o índice de lesões revelou diferenças significantes entre os participantes com e sem história de lesões no domínio peso. Os participantes que realizavam levantamento intenso de pesos, tiveram maior incidência de lesões em relação aos que realizavam levantamento de pesos de intensidade moderada ou leve (p=0,043) (Tabela 4). Não houve diferenças significativas nos domínios velocidade e frequência.

**Tabela 4:** Correlações entre a intensidade do treinamento de CrossFit e lesões

Intensidade do treinamento de CrossFit		Sem lesões (%)	Com lesões (%)	Valor de p
Qual a intensidade de seu treinamento? (peso)				0,043
	Leve ou moderado	56 (71,8)	23 (53,5)	
	Intenso	22 (28,2)	20 (46,5)	
Qual a intensidade do seu treinamento? (velocidade)				0,068
	Leve ou moderado	61 (78,2)	27 (62,8)	
	Intensão	17 (21,8)	16 (37,2)	
Qual a intensidade do seu treinamento? (frequência)				0,088
	Leve ou moderado	36 (46,2)	13 (30,2)	
	Intenso	42 (53,8)	30 (69,8)	

A maioria dos participantes (58,1%) que relataram lesões era do sexo masculino (p=0,018). Em média, os participantes com história de lesões dedicavam um tempo significativamente maior ao treinamento do que os sem história de lesões (68,4 e 61,7 minutos, respectivamente, p=0,044).

## DISCUSSÃO

O aumento substancial da popularidade do CrossFit trouxe maior preocupação com as lesões relacionadas à prática. Indivíduos sem experiência prévia em atividades físicas ou que se declaram sedentários também vêm aderindo a essa prática esportiva. Neste estudo, partiu-se da hipótese de que essas populações específicas seriam mais predispostas a lesões relacionadas ao CrossFit. Entretanto, os índices de lesões não diferiram de forma significante entre esses participantes e os fisicamente ativos. Visto que o CrossFit envolve movimentos complexos, a falta de condicionamento prévio é motivo óbvio de preocupação. De fato, os participantes com história de sedentarismo tiveram mais lesões do que os fisicamente ativos, porém as diferenças não foram significantes. Isso possivelmente se deveu ao tamanho pequeno da amostra. Ao comparar lesões relacionadas entre praticantes com história de sedentarismo e praticantes ativos, Sprey et al.,^(^^2^^)^ também encontraram índices muito semelhantes de lesões (30,4% e 31,2%, respectivamente). Um estudo prospectivo de coorte recente^(^^9^^)^ que investigou a incidência de lesões entre iniciantes matriculados em um programa de CrossFit de 8 semanas revelou risco mais elevado (14,9%) de lesões nessa população do que entre praticantes experientes.

Lesões relacionadas ao CrossFit decorrem principalmente da sobrecarga muscular durante a prática. Lesões por sobrecarga resultam de altos números de repetições de gestos esportivos, do levantamento de pesos que excedam a capacidade do praticante ou de qualquer outra atividade que ultrapasse seu limite fisiológico.^(^^11^^–^^13^^)^ Hopkins et al.,^(^^14^^)^ relatou 11 casos de rabdomiólise após a prática do CrossFit. Embora se acredite que o CrossFit esteja associado a uma maior incidência de lesões, estudos prévios revelaram incidências semelhantes às da corrida, do tênis, da ginástica e do levantamento de peso olímpico, além de uma incidência mais baixa em relação ao *rugby* e ao futebol.^(^^2^^–^^5^^,^^7^^,^^8^^)^ Assim como em outros estudos,^(^^2^^,^^5^^,^^15^^,^^16^^)^ a incidência de lesões neste estudo foi de 35,5%. Entretanto, estudo prospectivo recente revelou índice de 14,9%.^(^^9^^)^ Variações entre os índices de lesões relatados em diferentes estudos provavelmente refletem diferenças na definição de lesão adotada ou no perfil dos participantes.

A coluna, o ombro e o joelho são os locais mais comuns de lesões relacionadas ao CrossFit.^(^^5^^,^^7^^,^^8^^,^^14^^,^^15^^,^^17^^)^ Em estudo realizado por Hopkins et al., a coluna foi o local mais comum de lesões, respondendo por 20,9% do total de lesões (6,7% delas de resolução cirúrgica). O CrossFit envolve gestos esportivos semelhantes aos de atividades como ginástica e levantamento de peso e, portanto, as lesões são de padrão semelhante e ocorrem nos mesmo locais.^(^^14^^)^ Neste estudo, ombro/cotovelo foi o principal local de lesões, seguido da coluna (39,6% somando lombar e cervical) enquanto a incidência de lesões no joelho foi baixa (7%). Segundo Sugimoto et al.,^(^^18^^)^ as chances de lesões no membro inferior são 2,65 vezes mais altas em mulheres do que em homens. Em contrapartida, as lesões de ombro são 2,79 mais prováveis em homens, enquanto as de coluna afetam principalmente praticantes de menos de 19 anos de idade.

O sexo masculino, a prática por tempo prolongado, a intensidade do treinamento, a participação em competições, o IMC elevado, a falta de supervisão durante o treinamento e as lesões prévias são alguns dos fatores de risco para lesões relacionadas ao CrossFit,^(^^2^^,^^5^^–^^8^^)^ dados esses corroborados pelos achados deste estudo. Nesta amostra, o treinamento intenso em termos de frequência e peso mostrou-se associado a índices mais elevados de lesões, embora as diferenças significantes tenham se limitado ao domínio peso. Os participantes com história de lesões passavam mais tempo treinando (68,4 minutos) do que os que não relataram lesões. Além disso, a ocorrência de lesões foi mais comum em homens do que em mulheres.

A principal limitação deste estudo foi o tamanho relativamente pequeno da amostra para análise estatística. Isso provavelmente refletiu a relutância de alguns ginásios de CrossFit em participar desta pesquisa e compartilhar o questionário entre seus atletas. O desenho transversal foi outra limitação, principalmente devido à percepção subjetiva do que de fato constitui uma lesão entre os respondentes, embora a definição de lesão tenha sido reforçada no questionário. Dentre as vantagens dos questionários digitais, destacam-se a facilidade de distribuição e o fornecimento de cobertura abrangente para fins de pesquisa. Entretanto, eles estão sujeitos à interpretação dos respondentes.

## CONCLUSÃO

A incidência de lesões relacionadas ao CrossFit não diferiu de forma significante entre participantes com história de sedentarismo e praticantes fisicamente ativos. A prática intensa de levantamento de peso mostrou-se associada a uma maior incidência de lesões.
